# Super-resolution of magnetic systems using deep learning

**DOI:** 10.1038/s41598-023-38335-y

**Published:** 2023-07-17

**Authors:** D. B. Lee, H. G. Yoon, S. M. Park, J. W. Choi, G. Chen, H. Y. Kwon, C. Won

**Affiliations:** 1 Department of Physics, Kyung Hee University, Seoul, 02447 South Korea; 2 Department of Battery-Smart Factory, Korea University, Seoul, 02841 South Korea; 3 Center for Spintronics, Korea Institute of Science and Technology, Seoul, 02792 South Korea; 4 National Laboratory of Solid State Microstructures and Department of Physics, Nanjing University, Nanjing, 210093 China; 5 Collaborative Innovation Center of Advanced Microstructures, Nanjing, 210093 China

**Keywords:** Magnetic properties and materials, Imaging techniques

## Abstract

We construct a deep neural network to enhance the resolution of spin structure images formed by spontaneous symmetry breaking in the magnetic systems. Through the deep neural network, an image is expanded to a super-resolution image and reduced to the original image size to be fitted with the input feed image. The network does not require ground truth images in the training process. Therefore, it can be applied when low-resolution images are provided as training datasets, while high-resolution images are not obtainable due to the intrinsic limitation of microscope techniques. To show the usefulness of the network, we train the network with two types of simulated magnetic structure images; one is from self-organized maze patterns made of chiral magnetic structures, and the other is from magnetic domains separated by walls that are topological defects of the system. The network successfully generates high-resolution images highly correlated with the exact solutions in both cases. To investigate the effectiveness and the differences between datasets, we study the network’s noise tolerance and compare the networks’ reliabilities. The network is applied with experimental data obtained by magneto-optical Kerr effect microscopy and spin-polarized low-energy electron microscopy.

## Introduction

Computational approaches have been extensively applied to study various scientific systems. As well as the numerical simulation, one of the representative conventional methods widely performed to understand the physical properties of the systems, deep learning techniques based on deep artificial neural networks have been adopted as a novel and innovative computational approach recently. For example, deep learning techniques are used to solve many-body problems^1–4^ and to explore phase transitions^5–8^ in various physical systems.

In magnetism studies, deep learning techniques are also effectively applied to investigate the physical properties of magnetic systems. In magnetic systems, the spin Hamiltonian governing the physics of the systems includes various energy terms, and the competition between the energy terms induces interesting magnetic properties. Specifically, it is well known that unique magnetic structures, such as the magnetic stripe domains^9–11^ and magnetic skyrmions^12–15^, can appear on various magnetic systems. These magnetic structures have been intensively studied using not only conventional micro-magnetic simulation techniques^16–19^ but also numerical approaches based on deep learning techniques^20–24^ for applications to new spin devices.

To reveal the existence of magnetic structures and to investigate their physical properties, experimental observations using microscopy techniques are usually used in most of the experimental studies in magnetism literature. However, it is often difficult to obtain high-resolution images of magnetic domains due to the physical limitations of the experimental equipment. This limitation may disturb the quantitative and detailed analysis of the magnetic structures from the experimentally observed images. Therefore, it is expected that enhancing the resolution of raw images will bring significant advances in understanding the physical properties of magnetic systems.

In this situation, image resolution enhancement technology using deep learning^25–28^, called a super-resolution (SR) technique, has emerged and been applied to various scientific research fields. In particular, several studies to enhance the resolution of images from scanning electron microscopy, electron backscatter diffraction, and atomic force microscopy have been conducted using deep learning techniques based on convolutional neural network (CNN), generative adversarial network, and residual network^29–35^. The conventional SR techniques based on deep learning require high-resolution data as the target of the supervised learning. However, high-resolution data may be hardly obtainable or even not be available.

In this study, we present an unsupervised deep learning technique to produce the SR data from low-resolution (LR) spin configuration data. We construct a deep neural network structure using multiple CNN layers, referred to as an SR network in this study. It is designed to require only the LR images for the training process, and training proceeds through unsupervised learning.

To check the effectiveness of our approach, first, we train the SR network using the simulated spin configuration images. Using a Monte Carlo simulated annealing method, we generate two different types of spin configuration datasets, one is for the spin configurations composed of magnetic labyrinth textures, and the other is for the spin configurations composed of alternating out-of-plane magnetic domains with thin magnetic domain walls. These two datasets are separately used to train two identically structured SR networks. After the training process, we confirm that the trained networks can properly produce SR data and the capability of our approach certainly exceeds the conventional upscaling algorithms based on usual interpolation methods. We explain that SR networks are trained to catch the physical properties of each dataset by cross-feeding datasets and checking noise response. Finally, we apply the method to the magnetic domain images obtained by magneto-optical Kerr effect microscopy and spin-polarized low-energy electron microscopy.

## Results

### Super-resolution network

**Figure 1:**
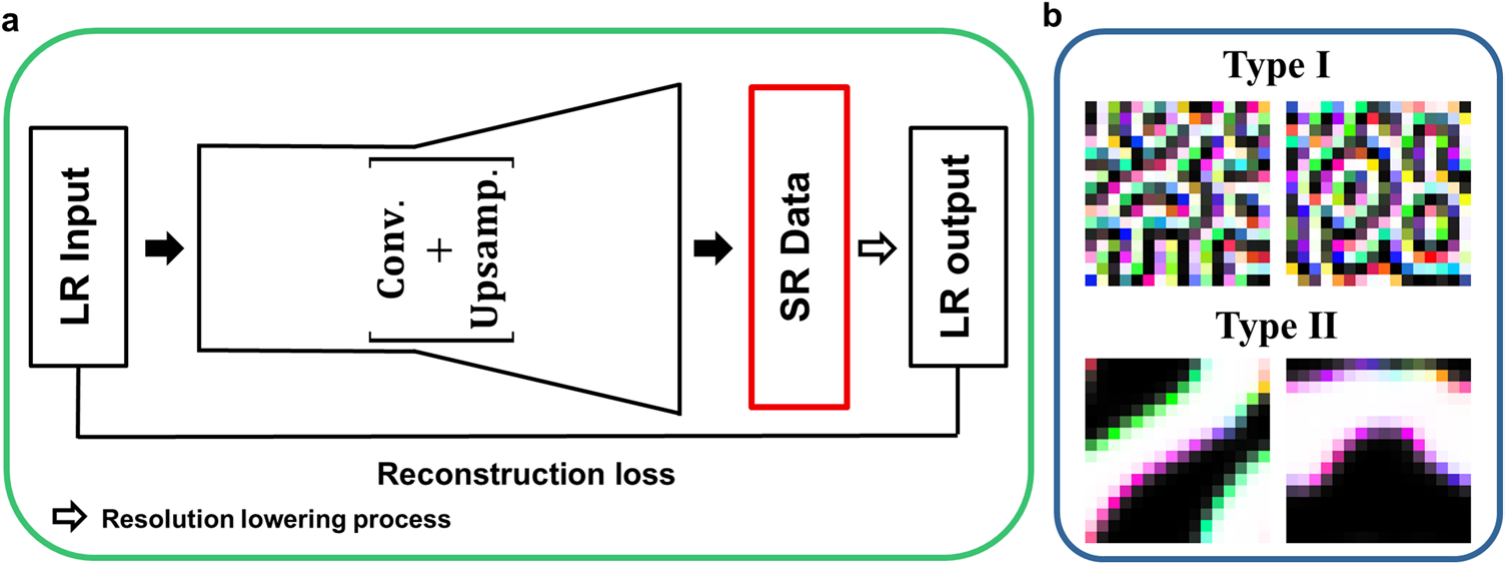
Super-resolution network structure and training dataset. (**a**) Schematic diagram of the Super-resolution network structure used in this study. LR, SR, Conv., and Upsamp. indicate low-resolution, super-resolution, convolutional neural network, and upsampling layer, respectively. (**b**) Sample images from two types of data. Type I and II data are LR samples of spin configurations composed of (I) labyrinth textures and (II) large domains with thin domain walls. For more details, see the “Experimental section”.

Our purpose is to enhance the resolution of the input image using deep learning techniques in an unsupervised manner without involving high-resolution images in the training process. Since it is often impossible to secure the high-resolution image dataset in experiments, for example, due to the limitation of resolution power, it is desirable to enhance the resolution of LR images without the high-resolution data. To achieve this goal, we construct the SR network according to the workflow shown in Fig. 1a.

The SR network is composed of two main parts. One is an up-sampling deep neural network composed of several CNN and simple up-sampling layers. This network transforms LR input data into SR data with a larger image size. The other is the resolution-lowering process from the SR data to the LR output. In this process, we use a Gaussian kernel down-sampling method that combines Gaussian blurring and average pooling. One should consider the original limitation causing low-resolution images to choose the resolution-lowering process used in the algorithm. Depending on the reason, for example, diffraction limit or thermal noise, one can determine the down-sampling process, thus, the process is not unique. One can choose other down-sampling methods which contain the Gaussian blurring, noise addition, resolution dropping, or mosaic processing to use a similar deterioration mechanism occurring in actual data acquisition. Note that the resolution-lowering process is chosen and fixed, not altered by training. Detailed network architecture and Gaussian kernel down-sampling process are described in the “Experimental section”.

We train the SR network to minimize the reconstruction loss between the LR input data and LR output data, as shown in Fig. 1a; using a well-trained SR network, the LR input fed into the network can be reproduced exactly as the LR output. Through the training process, we expect that the SR data that are the output of the deep neural network become the high-resolution images corresponding to the LR input images because the LR output results from a deterministic down-sampling from the SR data.

### Simulated spin configuration dataset

To verify the effectiveness of our approach, first, we train the SR network using the simulated spin configuration dataset. We generate spin configurations (high-resolution ground truth images) formed on a $128\times 128$ square grid system using a simulated annealing method implemented by a Monte-Carlo method which is used in several previous studies to simulate the magnetic structures appearing on magnetic systems^18,19,36^.

The spin configuration dataset comprises two different types of spin texture, referred to as Type I and Type II, in this study. Type I is for self-organized structures where similar patterns cover the space. We use the labyrinth texture that shows randomly oriented patterns with a constant structural length scale and chiral spin ordering. It is known that this type of structure originates mainly from the competition between the exchange interaction and Dzyaloshinskii-Moriya interaction (DMI) which is commonly considered in the studies about the chiral magnetic structures appearing on the systems with broken inversion symmetry^18,19,36^. Type II is for topological defect structure between domains formed by spontaneous symmetry breaking. We use the magnetic domains of locally uniform magnetization separated by domain walls. The characteristics of the domain and domain walls are determined by the exchange interaction, perpendicular magnetic anisotropy, and magnetic dipole–dipole interaction^11,37–40^. Details of the data generation process, Hamiltonian, and physical parameters are described in the “Experimental section”.

To obtain the LR datasets, as shown in Fig. 1b, we apply a typical downsizing method based on a Gaussian filter to the simulated spin configurations; the original $128\times 128$ image size of the simulated spin configuration is reduced to a smaller size such as $32\times 32$, $16\times 16$, or $8\times 8$. In this study, the term “$\times \mathrm{N}$” is used to represent the scale of resolution enhancement when the SR layer is constructed to produce $\mathrm{Na}\times \mathrm{Na}$ ($=128\times 128$) size image to increase the resolution of the LR image with $\mathrm{a}\times \mathrm{a}$ size. We have prepared a dataset containing 40,100 LR images of Type I and 40,100 LR images of Type II. Out of them, 40,000 images are used as training dataset, and 100 images from each type are used as test dataset. The detailed information of the dataset is described in the “Experimental section”.

### SR network performances for Type I and II datasets

We train our SR networks using the LR datasets until they properly reconstruct the input LR images. In this study, the SR networks trained with Type I and II are called *Net*. I and II, respectively. We compare the performances of *Net*. I and II with simple conventional resolution enhancing methods, such as bilinear, and bicubic interpolation. For quantitative analysis, we use several metrics measuring the image similarity typically considered in usual SR literature, such as mean square error (MSE), peak signal-to-noise ratio (PSNR), and spin–spin correlation (Corr.) between the ground truth and SR results. The details of how to calculate the spin configuration similarity are described in the “Experimental section”.

#### *Net*. I

**Figure 2:**
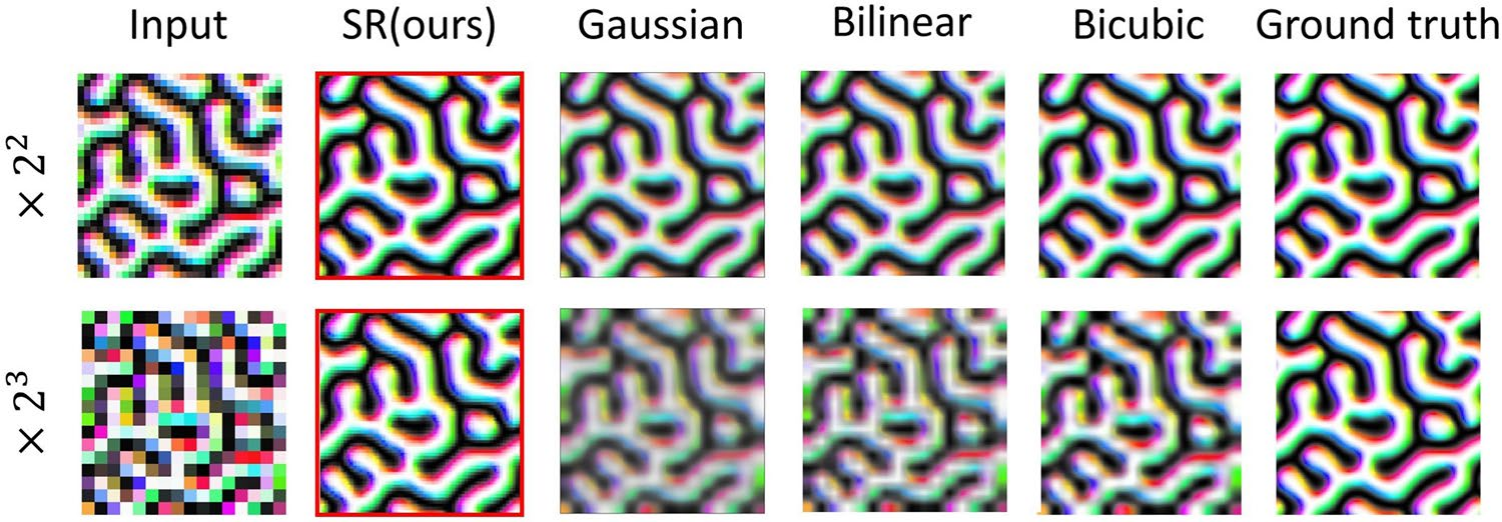
Results of $\times {2}^{2}$ and $\times {2}^{3}$ processes with Type I test dataset. The first column shows input images. The second column with images in red boxes is from the SR layer of our network. The following two columns show simple interpolation methods (Gaussian, bilinear, and bicubic), and the final one is the ground truth images from the original simulation result.

We investigate the performances of *Net*. I trained with $32\times 32$ and $16\times 16$ LR images, and compare them with the results of simple interpolation methods and ground truth simulation images as shown in Fig. 2.

The red-boxed images are the results from our network's SR layer, surpassing other conventional interpolation methods. Indeed, they are very close to the ground truth images both in $\times {2}^{2}$ and $\times {2}^{3}$ processes. Especially in the $\times {2}^{3}$ process, while the simple interpolation methods cannot properly generate high-resolution images, our SR network generates high-quality SR images from the degraded quality of the input image.

We calculate the MSE, PSNR, and Corr. for 100 test data for more quantitative analysis, as shown in Table 1. Our SR network results show superior results compared with the Gaussian kernel up-sampling. It indicates the trained up-scaling method in our network is not simple Gaussian up-scaling, though we use Gaussian kernel down-sampling from SR layer to LR layer. Our SR network shows the smallest value in MSE, the highest value in PSNR, and the closest to 1 in Corr., regardless of the SR ratios. These results indicate that our SR network produces highly reliable SR images from LR images, surpassing other simple interpolation methods.

**Table 1:** Performance table. MSE, PSNR, and Corr. are calculated for the SR network with 100 Type I test data and compared with other simple methods.

Ratio	Metric	Gaussian	Bilinear	Bicubic	SR network (ours)
$\times {2}^{2}$	MSE	0.64	$0.062$	$0.061$	$0.0016$
	PSNR	$24.00 \pm 0.20$	$24.14\pm 0.24$	$24.22\pm 0.30$	$39.96\pm 0.02$
	Corr	0.74	$0.77$	$0.85$	$0.9976$
$\times {2}^{3}$	MSE	0.236	$0.243$	$0.262$	$0.0065$
	PSNR	18.33 $\pm$ 0.13	$18.20\pm 0.18$	$17.89\pm 0.26$	$33.96\pm 0.03$
	Corr	0.301	$0.353$	$0.44$	$0.99$

#### *Net*. II

Unlike Type I data, Type II has no unique periodicity for domain width and no global chirality in spin configuration but shows wide domain areas separated by domain walls. Thus, it is a more challenging problem for our SR network. The SR network is trained on the data lowered to $32\times 32$, $16\times 16$, and $8\times 8$ using the Gaussian filter downsizing from the ground truth data with a size of $128\times 128$.

**Figure 3:**
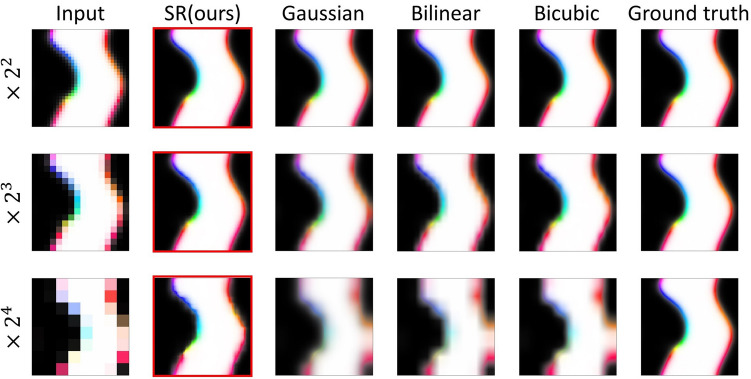
Results of $\times {2}^{2}$, $\times {2}^{3}$, and $\times {2}^{4}$ processes with Type II test data. Inputs, SR network results, Gaussian, bilinear, bicubic, and ground truth simulation images are compared.

Figure 3 shows the results when images of $32\times 32$, $16\times 16$, and $8\times 8$ pixel data are fed into an SR network and the results from simple interpolation methods (Gaussian, bilinear, and bicubic). We confirm that the domain walls shown in our SR results are more precise than other interpolation methods. In the result of the $\times {2}^{4}$ process, the ground truth and the result of our network are slightly different. However, it still shows the most physically plausible result than other simple interpolations.

The quantitative analysis for Type II data is presented in Table 2 with MSE, PSNR, and Corr. analysis between ground truth and upscaling results with 100 test data. Our SR network shows the smallest value in MSE, the highest value in PSNR, and the closest value to 1 in the Corr. of all methods.

**Table 2:** The performance table (MSE, PSNR, and Corr.) for the SR network for 100 Type II test data.

Ratio	Metric	Gaussian	Bilinear	Bicubic	SR network (ours)
$\times {2}^{2}$	MSE	0.0060	$0.0059$	$0.0058$	$0.00018$
	PSNR	34.49 ± 2.76	$34.60\pm 3.01$	$34.70\pm 3.56$	$49.68\pm 1.2$
	Corr	0.974	$0.978$	$0.986$	$0.9997$
$\times {2}^{3}$	MSE	0.027	$0.028$	$0.029$	$0.00046$
	PSNR	$27.90\pm 2.67$	$27.90\pm 2.91$	$27.81\pm 3.37$	$45.68\pm 1.13$
	Corr	0.91	$0.92$	$0.94$	$0.999$
$\times {2}^{4}$	MSE	0.0883	$0.0913$	$0.0975$	$0.0061$
	PSNR	$22.80\pm 2.63$	$22.66\pm 2.79$	$22.41\pm 3.02$	$34.45\pm 2.24$
	Corr	0.76	$0.78$	$0.82$	$0.99$

### Cross-feeding data to the trained networks with Type I and Type II

The networks trained by Type I and Type II datasets show excellent performance in increasing resolution. In this section, we explain that our SR network increases the resolution not only with a general high-order interpolation but also using specific properties of input data obtained during the training process. To verify that our network has trained the basic physical characteristics of images, we perform cross-feeding data by feeding Type II as an input to *Net*. I, denoted as *Net*. I (Type II), and Type II as an input to *Net.* II, denoted as *Net.* II (Type I).

**Figure 4:**
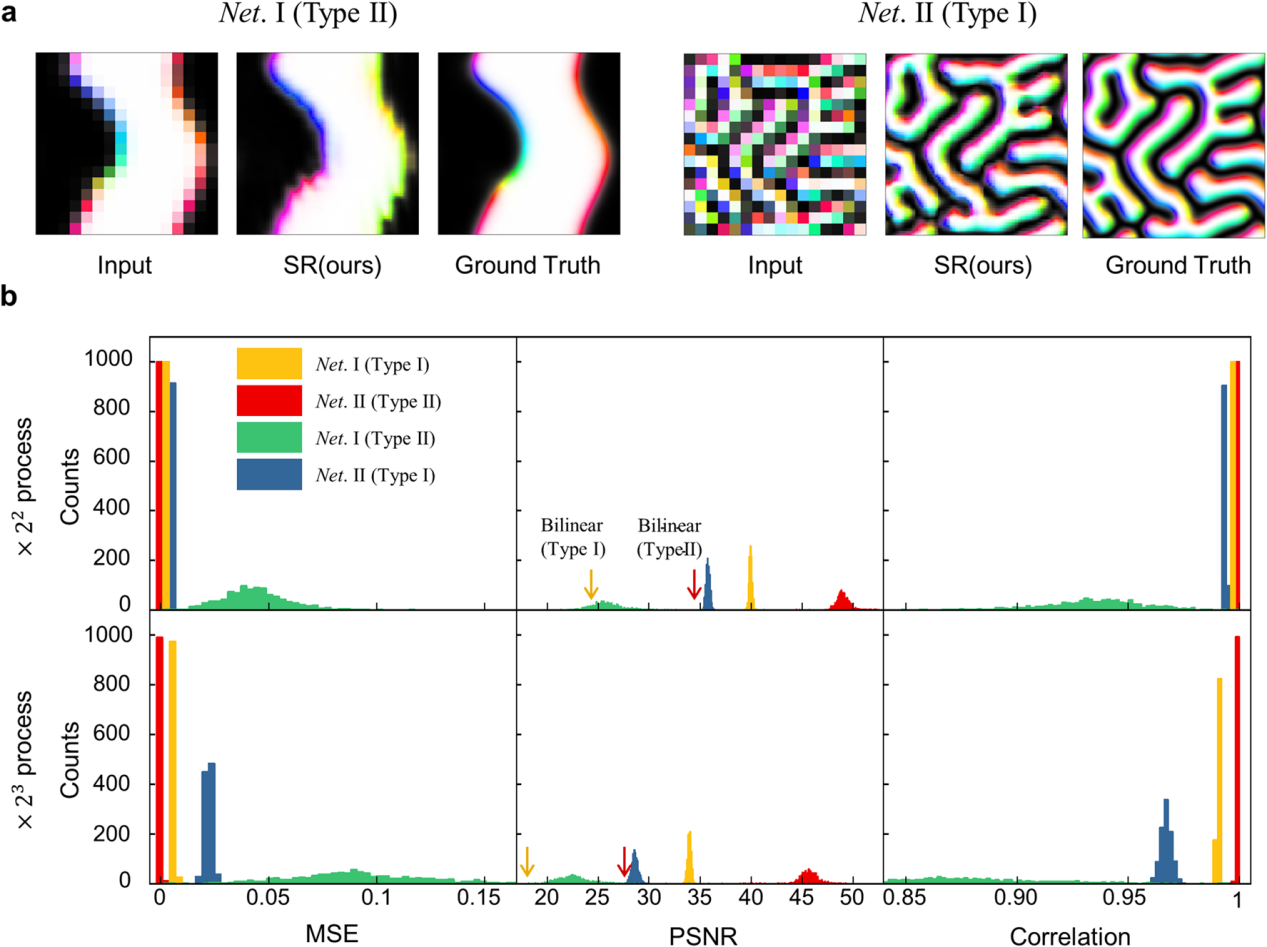
Analysis for cross-feeding different types of spin configuration data. (**a**) Input, SR result, and ground truth data of the spin configurations for *Net*. I (Type II) and *Net*. II (Type I). (**b**) The distributions of MSE, PSNR, and Corr. of *Net*. I (Type I), *Net*. II (Type II), *Net*. I (Type II) and *Net*. I (Type II) of 100 test data. The yellow and red arrows of PSNR represent the average value of the bilinear interpolation process for Type I and Type II, respectively.

Figure 4a shows the spin configurations of the input data of Type I and Type II in the case of cross-feeding and compares them with the ground truth. When *Net*. I is fed with Type II, it converts the spin direction following the chirality rule trained with Type I dataset, though it is not correct for Type II data. When *Net*. II is fed Type I, the results are close to the ground truth, thus showing good improvement of image resolution, though the quality is not better than the case of *Net*. I.

We compare statistically how the performance is lowered when the data is cross-fed. Figure 4b shows graphs for $\times {2}^{2}$ process and $\times {2}^{3}$ process from four cases, *Net*. I with Type I dataset, *Net*. I with Type II dataset, *Net*. II with Type I dataset, and *Net*. II with Type II dataset. We confirm that high performance is shown in the order of *Net.* II (Type II), *Net.* I (Type I), *Net.* II (Type I), and *Net.* I (Type II) of the four cases. The cross-feeding results are still better than the simple interpolation results, which means the SR network trains the general rule of resolution enhancement by interpolation whether it is trained by either Type I or Type II data. However, the network also catches the exclusive characteristics of the training dataset, thus, the performance is lowered when it is applied to a different dataset. For example, when *Net*. I is applied to Type II, it applies the unique chirality rule on the Type II data, which originally do not have the rule and the results of *Net.* I (Type II) have higher MSE, lower PSNR, and lower Corr. than those of *Net.* II (Type II), as shown in Fig. 4.

### Noise response

Two datasets have a distinctive difference in the distribution of structural information in the images. Type I dataset contains chiral patterns in which the direction of spin continuously changes, and the structural information is distributed evenly in the image. In contrast, the Type II dataset contains topological defects, and the information is mostly concentrated in the shallow area, thus constructing an SR image from the Type II is a more subtle and difficult problem. To check the stability of the networks and their capability to handle nonuniformity, the networks are tested with noise-injected data. There are extensive studies of denoising with deep learning networks^23,41–43^ and generating data similar to trained data from random data^23^. We add noise to the spin configuration; ${\widehat{S}}{\prime}={L}_{2}(\widehat{S}+\alpha \widehat{N})$, where ${\widehat{S}}^{\prime}$ is a reconstructed new spin configuration with added noise, $\widehat{S}$ is the spin configuration before noise is added, $\widehat{N}$ is the added noise vector with a unit random vector map, $\alpha$ represents the intensity of the noise and ${L}_{2}$ is the L2-normalization process.

We use a recursive process that feeds the output data from the SR network back to the input data. The recursive process is performed for 100 iterations of each of the 100 Type I and Type II test data. and we analyze each data with various noise intensities.

**Figure 5:**
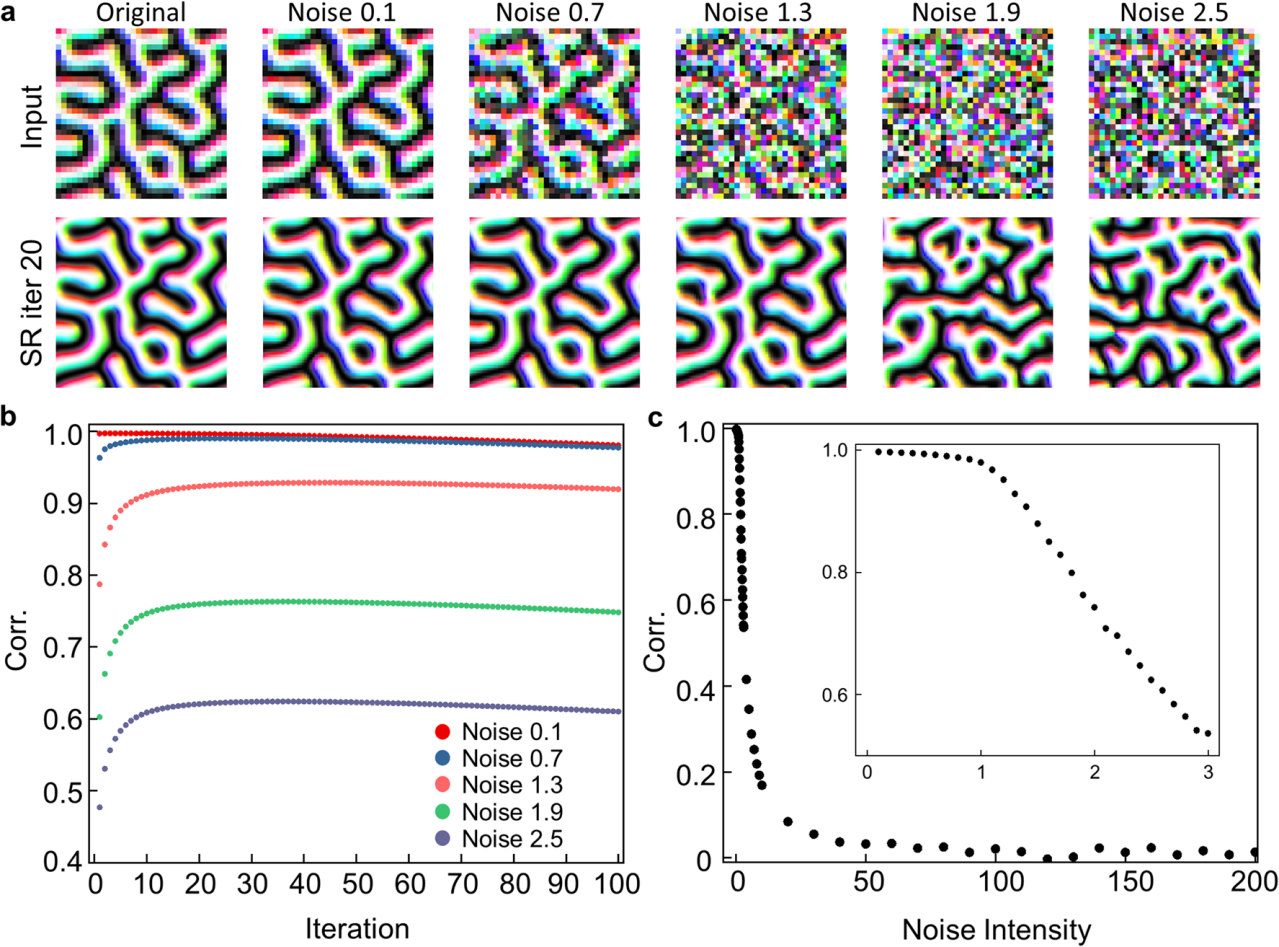
Analysis for noise tolerance in Type I data. (**a**) The original data and noise-added data (top layer), and corresponding results after the recursive process of 20 iterations (bottom layer). (**b**) Average values of Corr. for each iteration when the recursive process is progressed for 100 Type I test data. (**c**) The maximum values of Corr. according to the noise intensity after 100 recursive iterations. The inset figure is to magnify the low noise intensity region.

In the case of Type I, $\alpha$ is changed from 0 to 200 and the output spin configuration results according to the noise intensity are compared. Figure 5a shows the SR results when the SR network is fed to a recursive process with various noise levels. When the noise intensity is below 1, the noise is removed by the network, and the structure of the spin configuration preserves the input structure. When noise intensity exceeds 1, noise is also removed, but the spin configuration is not kept the same as the original structure.

We analyzed how much the original structures are transformed. Figure 5b shows the Corr. between the ground truth high-resolution spin configuration and the result of the SR spin configuration depending on the recursive process iteration. As the iteration increases, Corr. tends to increase with the first few iterations and decrease slowly thereafter, mostly around its maximum. It increases in the first several iterations because the noise is getting removed, but then it decreases slowly as the error accumulates while repeating the recursive process. As the noise intensity increases, the obtainable maximum of the Corr. decreases.

Figure 5c shows how the peak value changes according to the noise intensity. It decreases only slightly until the noise level is around 1.0 and it begins to decrease rapidly as the noise intensity increases above 1.0. Because the spin data is normalized, a noise intensity over 1.0 means it exceeds the signal and becomes sufficient to erase the original spin configuration information. At higher noise intensities, the noise intensity is so strong that the input spin configuration can be considered random. Even in this case, it generates a new spin configuration with the same characteristics as Type I. Therefore, it suggests that the SR network learns general features of the system even enough to be used as a generator.

**Figure 6:**
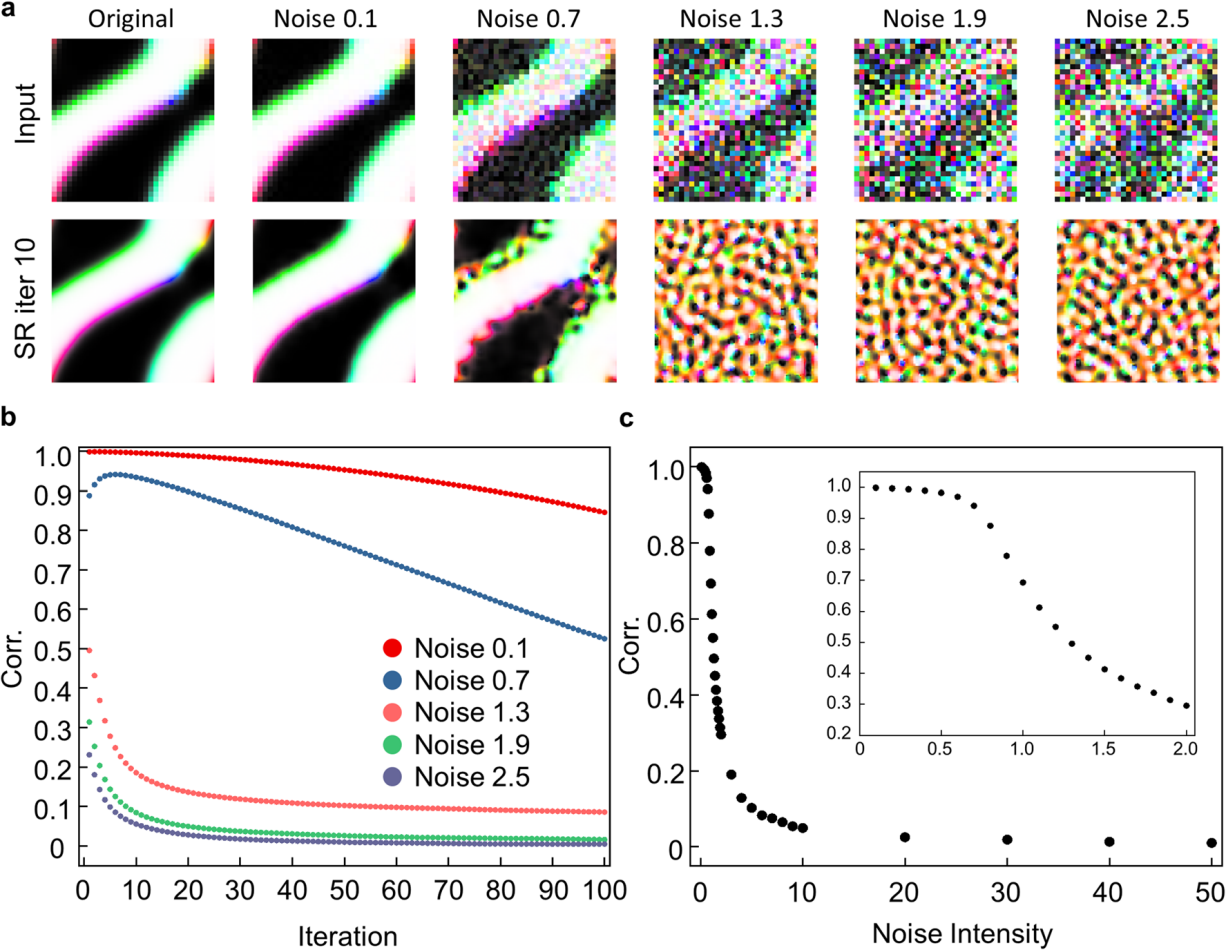
Analysis for noise tolerance in Type II data. (**a**) Input and output images for Type II dataset. The noise is increased by 0.6 from 0.1 to 2.5. and the results are shown after 10 recursive processes. (**b**) Average values of Corr. for each iteration. The recursive process is progressed for 100 Type II test data. (**c**) The maximum values of Corr. according to the noise intensity after 100 recursive iterations. The inset figure is to magnify the low noise intensity region.

In the case of Type II, $\alpha$ is changed from 0 to 50. Figure 6a shows the SR results when the SR network is fed to a recursive process without noise or with various noise levels. When the noise intensities are small, the SR network reduces the noise as in Type I. But as noise intensity is close to 1.0, the domain walls of the SR spin configuration are broken due to added noise. In SR results at relatively large noise, the large-size domains disappear, and only the small dotted structures remain which is very different from the original structure. Thus, we can see that the network trained on type II is sensitive and fragile to noise.

Figure 6b shows the statistical calculation results of the Corr. When noise intensities are less than 1.0, the Corr. initially increases during the first few iterations, then decreases rapidly. It initially increases by removing noise, similar to the result of Type I. However, it decreases rapidly at a further recursive process. When the noise intensities are higher, it only reduces with the recursive process, as the noise of the data cannot be removed and the domains are broken. The type II magnetic structures, unlike type I, contain the topological defect, or domain wall, separated by domains. The added noise tends to be interpreted as domain walls, thus, extensive noise extends defect structures and may weaken the reliability of SR networks, which suggests that the interpretation of SR results in practice should be careful when the system has defects or consists of various length-scale structures. Figure 6c shows the trend of the maximum value of the Corr. as a function of noise intensity. The Corr. decreases more sharply in Type II than in Type I, because Type II is more sensitive to noise than type I.

### Application to the SR network on the experimental data

In order to demonstrate the advantage of our SR network, that is, converting low-resolution data into super-resolution data without the target, we apply the network to real experimental data. Several microscopy techniques, such as magneto-optical Kerr effect (MOKE) microscopy^44–46^, scanning transmission X-ray microscopy (STXM)^14,47,48^, spin-polarized low-energy electron microscopy (SPLEEM)^37,38,40,49,50^, are used to obtain magnetic domain images. These microscopy techniques have limitations in the resolution at which magnetic structures can be observed. Here, we show that the application of the SR network can convert relatively low-resolution MOKE and SPLEEM experimental magnetic domain images into high-resolution images.

**Figure 7:**
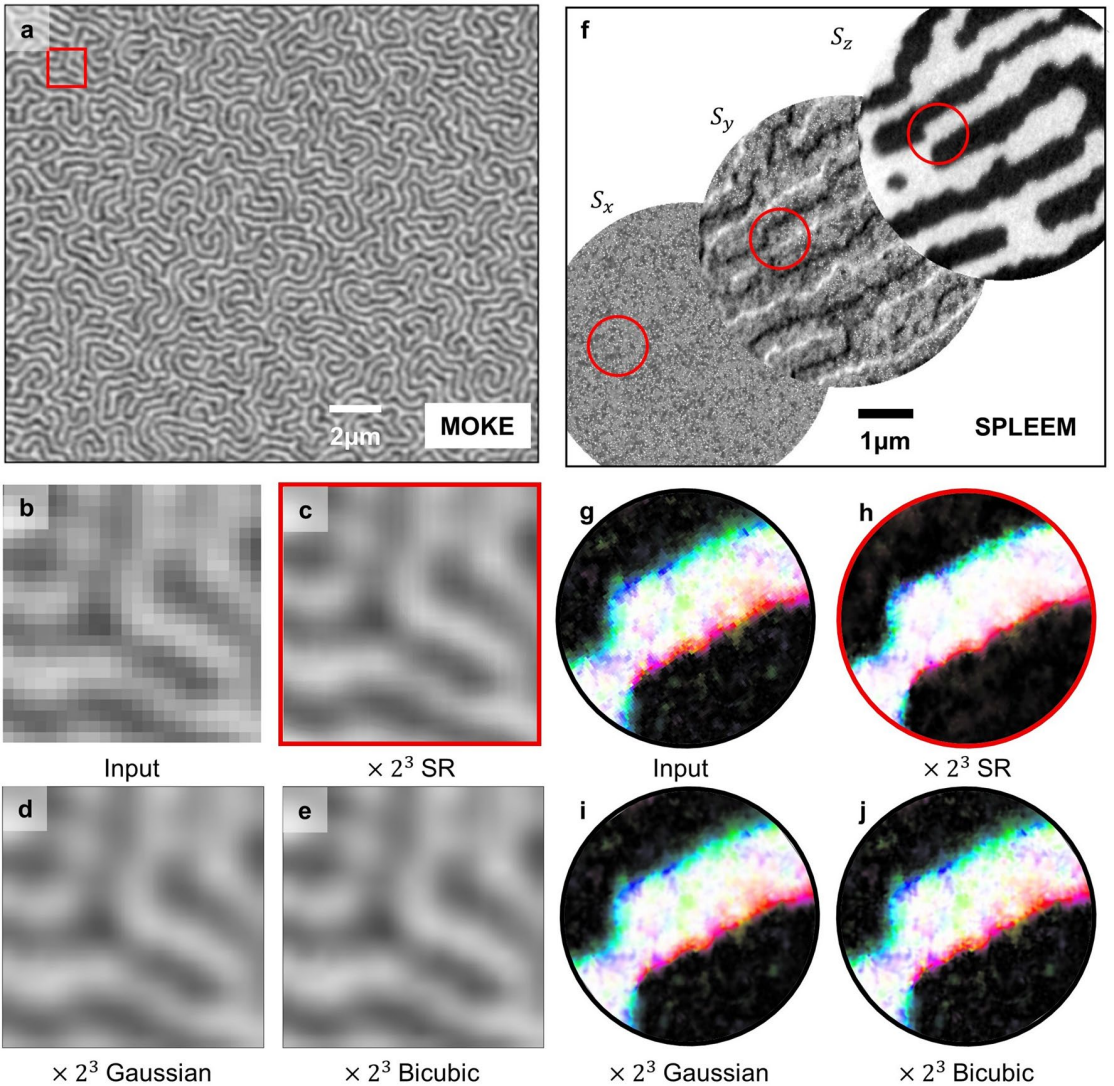
Producing high-resolution images using the SR network with the experimental data. (**a**) The spin structure from MOKE; scale bar indicates 2 μm. (**b**) The original MOKE data in the red box of (**a**). (**c**) The $\times {2}^{3}$ SR result from (**b**). (**d**,**e**) The $\times {2}^{3}$ Gaussian and bicubic interpolation results from (**b**). (**f**) The spin structure data from SPLEEM; scale bar indicates 1 μm. (**g**) The original resolution SPLEEM data in the red circle of (**f**). (**h**) The $\times {2}^{3}$ SR result from (**g**). (**i**,**j**) The $\times {2}^{3}$ Gaussian and bicubic interpolation results from (**g**). The parts we want to observe in detail are marked with red boundary line.

Figure 7a shows the MOKE microscopy data of the magnetic domain of a [Pt(3 nm)/GdFeCo(5 nm)/MgO(1 nm)]_20_ multilayer system. The images show complex labyrinth patterns which are similar to the Type I datasets. Detailed information about the magnetic domains in this material system is given in a previous study^14^. We split the Fig. 7a into 24,000 data and train the SR network using them. After training the SR network, we feed the MOKE data as input data to our SR network as shown in Fig. 7b, and the resulting converted $\times {2}^{3}$ data is shown in Fig. 7c. The original data shows blocks like a mosaic, but the resulting SR-network-applied data is a smooth image where the magnetic structure is more clear. The results obtained by other interpolation methods are shown in Fig. 7d,e for the comparison.

Figure 7f shows the experimental SPLEEM magnetic domain images of a Ni/Co/W system. The images show domains separated by domain walls, which are similar to the Type II datasets. Detailed information about the experimental environments and experimental systems is given in a previous study^40^. We concatenate Fig. 7f data to each of ${\mathrm{S}}_{\mathrm{x}}$, ${\mathrm{S}}_{\mathrm{y}}$, and ${\mathrm{S}}_{\mathrm{z}}$, normalize, split to 2800 data, and train the SR network using them. After training an SR network using the split data, we feed an image shown in Fig. 7g as the input, and the resulting $\times {2}^{3}$ data from the SR network is shown in Fig. 7h. The data is denoised due to the SR network, and the domain part and the domain wall part become more distinct. Except the noise reduction, the SR results do not show a significant difference, when they are compared to other interpolation methods (Fig. 7i,j).

The results cannot be compared with the true high-resolution data or prove its superiority to other conventional interpolation methods because the images do not have corresponding images with a higher magnification ratio. Nevertheless, our results show that low-resolution data can be converted to high-resolution data in practice by utilizing our SR network in an environment where high-resolution data is desirable but unobtainable. The SR network results depend on the resolution-lowering process in the network. We have used the same Gaussian kernel down-sampling used in the simulation dataset. The other down-sampling method better fitted with experimental situations may further enhance the SR results.

Our SR network has superiority compared with other deep learning techniques for SR^32–35^ because it does not require a high-resolution dataset in the training process. Most of the deep learning techniques use the high-resolution data as the target data of the supervised learning algorithm, thus it cannot be applied when the high-resolution data is not obtainable. In contrast, our SR network can be applied to LR data because it minimizes the reconstruction loss at the low-resolution level. Our approach shows that training without high-resolution data can efficiently provide correct results when the training data contained patterns based on the same physical origin. In these cases, the networks obtain global properties from the locally dispersed structural information, and SR images are constructed based on the properties.

## Conclusion

In this study, we devised SR networks using the deep learning method inferring super-resolution images without requiring high-resolution data in the training process. Our deep learning method has the potential to produce high-resolution images maintaining the physical properties of the target system. The trained networks with simulation data proved their utility with much-enhanced performance, compared with conventional interpolation methods. Through cross-feeding and noise response, we found that the networks were trained to follow the characteristics of the training dataset. Using this SR network for experimental data such as MOKE images and SPLEEM images, we confirmed the usability of our SR network in the experimental data. Our technique can be utilized in a lot of other scientific research areas where it is required to analyze and investigate image data characterizing physical states by converting low-resolution data into high-resolution.

## Experimental section

### Data generation

We choose the two types of two-dimensional magnetic spin configuration datasets generated under each Hamiltonian condition. The datasets are selected to evaluate the purpose of our SR network. Thus, the datasets should have various patterns with the same characteristics, and quantitative evaluations should be possible. The Type I dataset contains various patterns of labyrinth spin configurations, and the Type II dataset contains domains separated by domain walls.

To generate a Type I dataset, we use a square lattice Heisenberg spin model of two-dimensional magnetic systems with 128 × 128 size. Hamiltonian model, $\mathcal{H}=-J\sum_{<ij>}{\overrightarrow{S}}_{i}\cdot {\overrightarrow{S}}_{j}+\sum_{<ij>}{\overrightarrow{DM}}_{ij}\cdot \left({\overrightarrow{S}}_{i}\times {\overrightarrow{S}}_{j}\right),$ is used. $J$ is the exchange interaction parameter, $\overrightarrow{DM}$ is the Dzyaloshinskii-Moriya interaction vector, $\overrightarrow{S}$ is a normalized spin vector, and $i$ and $j$ represent grid sites. The length scale of the spin structure is determined by the ratio of $J$ and $|\overrightarrow{DM}|$ and we choose $J=1$ and $\left|{\overrightarrow{DM}}_{ij}\right|=0.3$ to have enough spin structure in the images.

The Type II dataset is also generated with a square lattice Heisenberg spin model of two-dimensional magnetic systems with 128 × 128 size. A simple Hamiltonian model is used; $\mathcal{H}=-J\sum_{<ij>}{\overrightarrow{S}}_{i}\cdot {\overrightarrow{S}}_{j}-D\sum_{<ij>}\frac{\left({\overrightarrow{S}}_{i}\cdot {\overrightarrow{r}}_{ij}\right)\left({\overrightarrow{S}}_{j}\cdot {\overrightarrow{r}}_{ij}\right)-{\overrightarrow{S}}_{i}\cdot {\overrightarrow{S}}_{j}{\left|{\overrightarrow{r}}_{ij}\right|}^{2}}{{\left|{\overrightarrow{r}}_{ij}\right|}^{5}}-K\sum_{i}{\mathrm{S}}_{i,z}^{2}$. $D$ is the magnetic dipolar interaction strength, ${\overrightarrow{r}}_{ij}$ is the displacement vector between the $i$ and $j$ sites, and $K$ is the magnetic anisotropy strength. To have the sizeable magnetic domain separated by the magnetic domain walls, the condition for small effective anisotropy is used. We choose $J=1$, $D=0.03,$ and $K=0.215$.

Many studies have been done on both types of systems, and we generated the spin configurations with a simulated annealing process based on those studies. Since the magnetic system temperature is gradually decreased from above the Curie temperature to 0, various metastable states are generated. We use this process to generate the 40,100 datasets for Type I and Type II each. Of each data type, 40,000 are used as training data, and 100 are used as test data.

### Data preprocessing: Gaussian filter downsizing

Periodic padding processes are performed on input data [128, 128, 3] size, $\frac{\mathrm{input \; data \; size}}{\mathrm{output \; data \;size}}$ is used as stride, and data are downsized by convolution with a Gaussian filter. In this study, the size of the Gaussian filter is [23, 23], and the sigma value of the Gaussian filter is 1. Through the Gaussian filter downsizing process, input data of $128\times 128$ size are converted into $32\times 32$ size, $16\times 16$ size, and $8\times 8$ size as needed.

### SR network structure

This study aims to devise a neural network for super-resolution spin configuration from low-resolution spin configuration using deep learning. We construct a convolutional neural network structure to obtain the low-resolution spin configuration from the same size input spin configuration. The SR spin configuration we ultimately want to obtain is a structure from the SR layer in front of the output layer. The network structure is constructed with two parts: expanding filter and a decoder. The expanding filter part, composed of four Convolutional Neural Network (CNN) layers with 64 filters, increases the input spin configuration of the three-dimensional vector map to 64 filters with $3\times 3$ filter sizes. Since our training spin configuration datasets are generated by satisfying the periodic boundary condition, we add a periodic padding process before all CNN layers to train the network under the same conditions. The batch normalization layer and the leaky rectified linear unit (Leaky ReLU) activation layer are constructed after all CNN layers. The decoder part decodes the high-dimensional filter map into the SR spin configuration. It consists of the number of up-sampling blocks for each ratio. The single up-sampling block comprises the periodic padding process, CNN layer with $3\times 3$ filter sizes, batch normalization layer, leaky ReLU activation layer, and an up-sampling layer of a $2\times 2$ filter. The up-sampling layer of the $2\times 2$ filter doubles the data size horizontally and vertically. So $\times {2}^{n}$ process ($n$ is an integer) is constructed of $n$ upsampling blocks. According to $\times {2}^{2}$, $\times {2}^{3}$, and $\times {2}^{4}$, the number of filters for the CNN layers are (16 and 8), (32, 16 and 8), and (48, 32, 16 and 8). After the up-sampling blocks, we add a periodic padding process and one CNN layer with three filters. The input and output data dimensions are the same. The input data are the Type I and Type II spin configurations generated under the two different Hamiltonians described above. The output data are two-dimensional spin configuration data composed of three-dimensional vectors reconstructed from the SR network.

We want to train the network structure so that the SR data are transformed into low-resolution spin configuration through Gaussian filter downsizing, and the output is a vector map. Therefore, we use the MSE $\langle {(\overrightarrow{S} -{\overrightarrow{S}}^{\prime})}^{2}\rangle$ as the total loss function. We train the SR network to lower the total loss. We verify our network's suitability during the SR network training by calculating the super-resolution validation loss. The validation loss is calculated from the MSE value between the ground truth data and the SR data, and validation loss is not used for training. Minimizing the total loss means that the output vector map approximates the input spin configuration and minimizing the validation loss means that the SR data approximates the correct ground truth data; therefore, after the training SR network, we can effectively estimate the SR data from the low-resolution data. We adopt the Adam optimizer^51^ to minimize the total loss, and its learning rate is fixed at 0.01.

### Spin configuration similarity measurement

We use well-known methods such as MSE, PSNR, and Corr. to quantitatively evaluate the similarity between ground truth and SR data from low-resolution test data. MSE is defined as $\frac{1}{mn}\sum_{i=0}^{m-1}\sum_{j=0}^{n-1}{\left[\overrightarrow{S}\left(i, j\right)-{\overrightarrow{S}}^{\prime}\left(i, j\right)\right]}^{2}$, where $\overrightarrow{S}$ and ${\overrightarrow{S}}^{\prime}$ represent ground truth spin configuration and reconstructed SR spin configuration respectively, and $i$ and $j$ represent the row and column number index of the grid site, respectively. PSNR is defined as $10{\mathrm{log}}_{10}\left(\frac{MA{X}^{2}}{MSE}\right)$, where $MAX$ is the maximum value. In our study, the spin values are represented from $-1$ to 1, and $MAX$ is equal to 2. Corr. is defined as $\frac{1}{mn}\sum_{i=0}^{m-1}\sum_{j=0}^{n-1}\overrightarrow{S}\left(i, j\right) \cdot {\overrightarrow{S}}{\prime}\left(i, j\right)$. Since the spin values ​​in our study range from $-1$ to 1, the values range from $-1$ to 1. When the value is 1, two spin data are entirely in the same state, and when it is 0, there is no relationship between them.

## Data Availability

The data used in the study is available from H. Y. K. and C. W. on reasonable request. The type I and II datasets used in this work are available on the following websites: https://data.mendeley.com/datasets/2vfnc426x3, https://data.mendeley.com/datasets/mhzydmtzzs.
